# TGF-βI Regulates Cell Migration through Pluripotent Transcription Factor OCT4 in Endometriosis

**DOI:** 10.1371/journal.pone.0145256

**Published:** 2015-12-16

**Authors:** Heng-Kien Au, Jui-Hung Chang, Yu-Chih Wu, Yung-Che Kuo, Yu-Hsi Chen, Wei-Chin Lee, Te-Sheng Chang, Pei-Chi Lan, Hung-Chih Kuo, Kha-Liang Lee, Mei-Tsu Lee, Chii-Ruey Tzeng, Yen-Hua Huang

**Affiliations:** 1 Department of Biochemistry and Molecular Cell Biology, School of Medicine, College of Medicine, Taipei Medical University, Taipei, Taiwan; 2 Graduate Institute of Medical Sciences, College of Medicine, Taipei Medical University, Taipei, Taiwan; 3 Center for Cell Therapy and Regeneration Medicine, Taipei Medical University, Taipei, Taiwan; 4 Department of Obstetrics and Gynecology, School of Medicine, College of Medicine, Taipei Medical University, Taipei, Taiwan; 5 Center for Reproductive Medicine, Taipei Medical University Hospital, Taipei Medical University, Taipei, Taiwan; 6 Department of Obstetrics and Gynecology, Taipei Medical University Hospital, Taipei, Taiwan; 7 Department of Gastroenterology and Hepatology, Chang Gung Memorial Hospital, Chiayi, Taiwan; 8 Institute of Cellular and Organismic Biology, Academia Sinica, Taipei, Taiwan; 9 Comprehensive Cancer Center of Taipei Medical University, Taipei, Taiwan; 10 The Ph.D. Program for Translational Medicine, Taipei Medical University, Taipei, Taiwan; Rutgers University, UNITED STATES

## Abstract

Transforming growth factor (TGF-β)/TGF-β receptor signal is known to promote cell migration. Up-regulation of TGF-β in serum/peritoneal fluid and increased levels of pluripotent transcription factor OCT4 in endometriotic tissues are frequently observed in patients with endometriosis. However, the mechanisms underlying how TGF-β/TGF-β receptor and OCT4 affect endometriotic cell migration still remain largely unknown. Therefore, endometriotic tissue with high cell migratory capacity were collected from patients with adenomyotic myometrium (*n* = 23) and chocolate cyst (*n* = 24); and endometrial tissue with low cell migratory capacity in normal endometrium or hyperplastic endometrium (*n* = 8) were collected as the controls. We found the mRNA levels of TGF-β receptor I (*TGF-β RI*) and *OCT4* were significantly higher in the high-migratory ectopic endometriotic tissues than those of the low-migratory normal or hyperplastic endometrium. Positive correlations between *TGF-β RI* and *OCT4*, and either *TGF-β RI* or *OCT4* with migration-related genes (*SNAIL*, *SLUG* and *TWIST*) regarding the mRNA levels were observed in human endometriotic tissues. TGF-βI dose-dependently increased the gene and protein levels of OCT4, SNAIL and N-Cadherin (N-CAD) and silencing of endogenous OCT4 significantly suppressed the TGF-βI-induced expressions of N-CAD and SNAIL in primary human endometriotic stromal cells and human endometrial carcinoma cell lines RL95-2 and HEC1A. Furthermore, TGF-βI significantly increased the migration ability of endometriotic cells and silencing of OCT4 dramatically suppressed the TGF-βI-induced cell migration activity evidenced by wound-closure assay, transwell assay, and confocal image of F-actin cellular distribution. In conclusion, the present findings demonstrate that the niche TGF-β plays a critical role in initiating expressions of pluripotent transcription factor OCT4 which may contribute to the ectopic endometrial growth by stimulating endometrial cell migration. These findings would be useful for developing therapeutic strategies targeting TGF-β-OCT4 signaling to prevent endometriosis in the future.

## Introduction

Endometriosis is a common chronic gynecological disorder defined as the presence of ectopic endometrial tissues, primarily on the pelvic peritoneum and the ovaries [1]. Several theories have been proposed regarding the pathogenesis of endometriosis [2–6]. It has been widely accepted that endometriosis originates from retrograde menstruation, wherein sloughed endometrial cells exit the uterus via the fallopian tubes [6]. Given the fact that regenerative capacity of the human endometrium is mediated by endometrial stem cells [7–9], recent studies have reported a correlation between aberrant stem-cell activity in the endometrium and endometriosis [10–14]. Several studies have reported increasing ectopic expression of stemness-related genes, including OCT4, in the endometria [15–17] and the upregulated OCT4 promoted the cell migration in human endometriosis [15]. However, the critical niche factors that regulate the OCT4 expression and cell migration in human endometriosis remain unclear.

Previous studies have indicated that certain niche substances may participate in the regulation of endometriosis, such as cytokines, chemokines, proteases, and growth factors [18–20]. The growth factors that promote endometrial cell migration, including the transforming growth factor-β (TGF-β), are critical in human endometriosis [19, 21, 22]. TGF-β is upregulated in several types of cancer [23–25]. TGF-β promotes cell migration and invasion in breast and lung cancers in a Smad-dependent manner through the induction of epithelial-to-mesenchymal transition (EMT) [26–31]; in addition, it increases the expression levels of TWIST, N-CADHERIN, and VIMENTIN in cholangiocarcinoma cells [32]. Moreover, TGF-β regulates various biological processes, including cell proliferation, differentiation, extracellular matrix formation, tissue remodeling, angiogenesis, immune response, inflammation, and apoptosis [33].

TGF-βI is among the most abnormally up-regulated growth factors found in the peritoneal fluid, endometriotic tissue, and serum [19, 21, 22]. It was hypothesized to contribute to the endometriosis development [34, 35]. As OCT4 is frequently up-regulated in endometriotic tissues [15–17], therefore, the present study aimed to investigate the roles of TGF-βI/TGF-β receptor (TGF-β R in human endometriosis by evaluating the expression profiles of OCT4 and TGF-β RI in ectopic endometrial tissues. Furthermore, we examined the role of TGF-βI in OCT4 expression and identified the biological influence of OCT4 by knocking down OCT4 expression during TGF-βI treatment using human primary endometrium stromal cell as the *in vitro* model. Our data indicated that TGF-βI initiates the expressions of pluripotent transcription factor OCT4, and OCT4 expression may be a crucial underlying molecular mechanism of TGF-βI-stimulated cell migration in human endometriosis.

## Materials and Methods

### Institutional approval and informed consent

All tissue samples were collected according to protocols that were approved by the TMU-Joint Institutional Review Board of Taipei Medical University (S1 Table). Written informed consent was obtained from all patients before the collection of tissue samples.

### Participants, tissue collection, and cell culture

Tissue samples of low-migratory capacity cells in normal endometrium and hyperplastic endometrium (*n* = 8), and of high-migratory capacity cells in adenomyotic myometrium (*n* = 23) and chocolate cyst (*n* = 24) tissue were collected by microdissection from patients undergoing laparoscopic tubal ligation or benign gynecological surgery at Taipei Medical University Hospital. Patients receiving hormone treatment and those with concurrent malignancies were excluded from our study. Human primary endometriotic stromal cells, provided by Dr. CR Tzeng at Taipei Medical University, Taipei, Taiwan, were generated from a single chocolate cyst tissue sample, as previously described [15]. RL95-2 and HEC1A human endometrial carcinoma cell lines (ATCC, Manassas, VA, USA) were cultured in Dulbecco’s modified Eagle’s medium (DMEM)/F12 (Gibco-BRL, Grand Island, NY, USA) with 10% fetal bovine serum (FBS, Gibco-BRL) at 37°C in 5% CO_2_ in a humidified incubator.

### RNA extraction, reverse transcription, and quantitative real-time PCR

All the tissue samples were frozen in liquid nitrogen immediately after collection. Primary endometriotic stromal cells were used as an *in vitro* cell model for cytokine/growth factor stimulation. The cells were treated with interleukin (IL)-6 (50 ng/mL), insulin-like growth factor I (IGF-I) (50 ng/mL), IL-1β (50 ng/mL), tumor necrosis factor (TNF)-α (50 ng/mL), and TGF-βI (1 ng/mL) for 24 hours. Total RNA was extracted using the RNeasy Micro Kit (Qiagen, Valencia, CA, USA), according to the manufacturer’s instructions. Complementary DNA (cDNA) was synthesized through reverse transcription (RT), using oligo-dT primers, 1.5 μg of total RNA, and Superscript III reverse transcriptase (Invitrogen, Carlsbad, CA, USA) according to the manufacturer’s instructions. Quantitative real-time PCR (qRT-PCR) was performed using the FastStart Universal SYBR Green Master Mix (Roche, Indianapolis, IN, USA) in a LightCycler 480 instrument (Roche), and the qRT-PCR results were recorded and analyzed using the instrument’s application software. This qRT-PCR method was used for mRNA analyses of all the genes evaluated in our study. The primer sequences used for TGF-β RI cDNA amplification flanked the nucleotide positions 6129 to 6206 (NM_004612) in the 5′ region of the TGF-β RI. The primer sequences used for *OCT4A* cDNA amplification flanked the nucleotide positions 369 to 516 (NM_002701) in the 5′ region of the *OCT4* coding sequence to distinguish the production of *OCT4B* cDNA. S2 Table summarizes the primer sequences used for amplifications. Beta-2 microglobulin expression was used to normalize the qRT-PCR results. The qRT-PCR assays were duplicated in three independent experiments for each experimental condition. The fold increase in gene expression was calculated relative to that of the RL95-2 cell line.

### Plasmids and transfection

The plasmids of shCtrl (TRCN0000072226), shOCT4#1 (TRCN00004879), and shOCT4#2 (TRCN000004881) were purchased from National RNAi Core, Taiwan. The RL95-2 and HEC1A cell lines and human primary endometriotic stromal cells were seeded in 6-well plates at densities of 4 × 10^5^, 2 × 10^5^, and 6 × 10^4^ cells/well, respectively, one day before cell transfection. The transfection mixture was prepared by diluting 4 μg of plasmid DNA and 3 μL of Turbofect reagent (Fermentas, Glen Burnie, MD, USA) in 500 μL of serum-free DMEM/F12 medium with gentle pipetting. Following incubation for 20 minutes, the transfection mixture was added to the culture medium and incubated for 6 hours. After starvation, the cells were treated with or without TGF-βI (1 ng/mL) for 24 hours.

### Western blotting

The transfected cells were lysed in an ice-cold protein lysis buffer containing 50 mM Tris, 150 mM NaCl, 5 mM EDTA, 0.1% sodium deoxycholate, and 1% sodium dodecylsulfate (SDS) (pH 8) that was supplemented with a protease inhibitor cocktail (Roche). The cells were incubated in the lysis buffer for 30 minutes on ice, followed by centrifugation at 15 000 ×*g* for 15 minutes. The supernatants were collected for western blot analyses. Cell lysate aliquots (30–100 μg of total protein) were subjected to SDS-polyacrylamide gel electrophoresis on a 10% acrylamide gel, and the protein bands were transferred to polyvinylidene difluoride membranes.

The primary antibodies (Abs) used for western blot analyses were as follows (S3 Table): (a) a rabbit anti-OCT4A monoclonal Ab (Epitomics, Burlingame, CA, USA) that specifically recognizes the N-terminus (amino acids 90–120) of the human OCT4A protein; (b) an anti-VIMENTIN Ab (Epitomics); (c) an anti-TWIST Ab (Santa Cruz Biotechnology, Santa Cruz, CA, USA); (d) an anti-SNAIL Ab (Cell Signaling Technology, Danvers, MA, USA); (e) an anti-N-CADHERIN Ab (Epitomics); and (g) an anti-Actin Ab (Sigma-Aldrich). A horseradish peroxidase-conjugated anti-mouse/rabbit Ab (Jackson ImmunoResearch, West Grove, PA, USA) was used as the secondary Ab, and the protein bands were visualized using an enhanced chemiluminescence system according to the manufacturer’s instructions (Millipore, Billerica, MA, USA).

### Wound-closure and transwell migration assays

For wound-closure assays, human primary endometriotic stromal cells were transfected with either the control vector or the shOCT4 plasmids and plated in 60-mm dishes at equivalent cell densities with or without TGF-βI treatment. Each cell monolayer was scratch wounded with a micropipette tip. After being washed with a medium to remove the detached cells, the adherent cells were incubated at 37°C in a 5% CO_2_ humidified atmosphere for 24 hours. Digital images of the scratch-wound area were acquired at each time point, and the gap area was measured.

Transwell assays were performed using 8-μm pore transwell chambers in 24-well plates (Corning Costar, Cambridge, MA, USA). The upper chambers were seeded with 1 × 10^5^ endometriotic stromal cells treated with or without TGF-βI in 100 μL of serum-free DMEM/F12 medium. The cells had been previously transfected with either the control vector or the shOCT4 plasmids. The lower chambers were filled with 800 μL DMEM/F12 medium containing 10% FBS. Subsequently, the cells were incubated at 37°C in a 5% CO_2_ humidified atmosphere for 24 hours. After swabbing the non-migrated cells from the upper chambers, the cells that had migrated to the lower chambers were fixed with 3.7% paraformaldehyde in phosphate-buffered saline and stained with hematoxylin. The cells that had migrated to the lower chambers were counted under a light microscope in 5 predetermined fields. The assay was performed in triplicate, and the results were expressed as the means of the cell percentages of the three wells.

### Immunocytochemistry

Human primary endometriotic stromal cells that were previously transfected with either the control vector or the shOCT4 plasmids were treated with or without TGF-βI (1 ng/mL). The cells were then fixed with 0.05% glutaraldehyde at room temperature for 15 minutes and blocked in 50 mg/mL of bovine serum albumin and 0.5% Triton X-100 in phosphate-buffered saline (pH 7) for 1 hour at room temperature. The blocked cells were incubated with an anti-Actin Ab (Sigma-Aldrich), and the bound primary Ab was detected with a FITC-conjugated secondary Ab (Jackson ImmunoResearch). The nuclei of the cells were counterstained with 4',6-diamidino-2-phenylindole (DAPI) (Sigma-Aldrich, St. Louis, MO, USA), and the cells were covered with an antifading reagent (Vector Laboratories, Burlingame, CA, USA) before examining them under a confocal-imaging fluorescence microscope (Leica, Buffalo Grove, IL, USA).

### Statistical analysis

All experiments were independently repeated in triplicate for each type of tissue, cell line, and primary cell. Results were expressed as the mean ± standard deviation (SD) for data with normal distribution, and the median and the interquartile range for data with non-normal distribution. The independent Student's *t*-test was used to compare continuous variables that followed a normal distribution, and Mann Whitney U test was used to compare those with non-normal distribution. The Chi-squared test was used to compare the categorical variables. The correlations between *TGF-β RI* and *OCT4* and either *TGF-β I* or *OCT4* with migration-related genes were calculated with the Pearson correlation analysis. A 2-tailed *P* value of < .05 was considered statistically significant. The GraphPad Prism 3.00 (GraphPad, San Diego, CA, USA) and Stata 11 for Windows (StataCorp LP, College Station, TX, USA) computer programs were used for statistical analyses.

## Results

### Expression profiles of pluripotent transcription factor *OCT4* and *TGF-β RI* in human endometrial tissues

The profiles of the study participants were presented in S1 Table. Compared with the control group of normal endometrium or hyperplasia, women with adenomyosis or chocolate cyst had a younger (*P* = .005) age and a higher level of cancer antigen (CA) 125 (*P* = .04).

**Fig 1:**
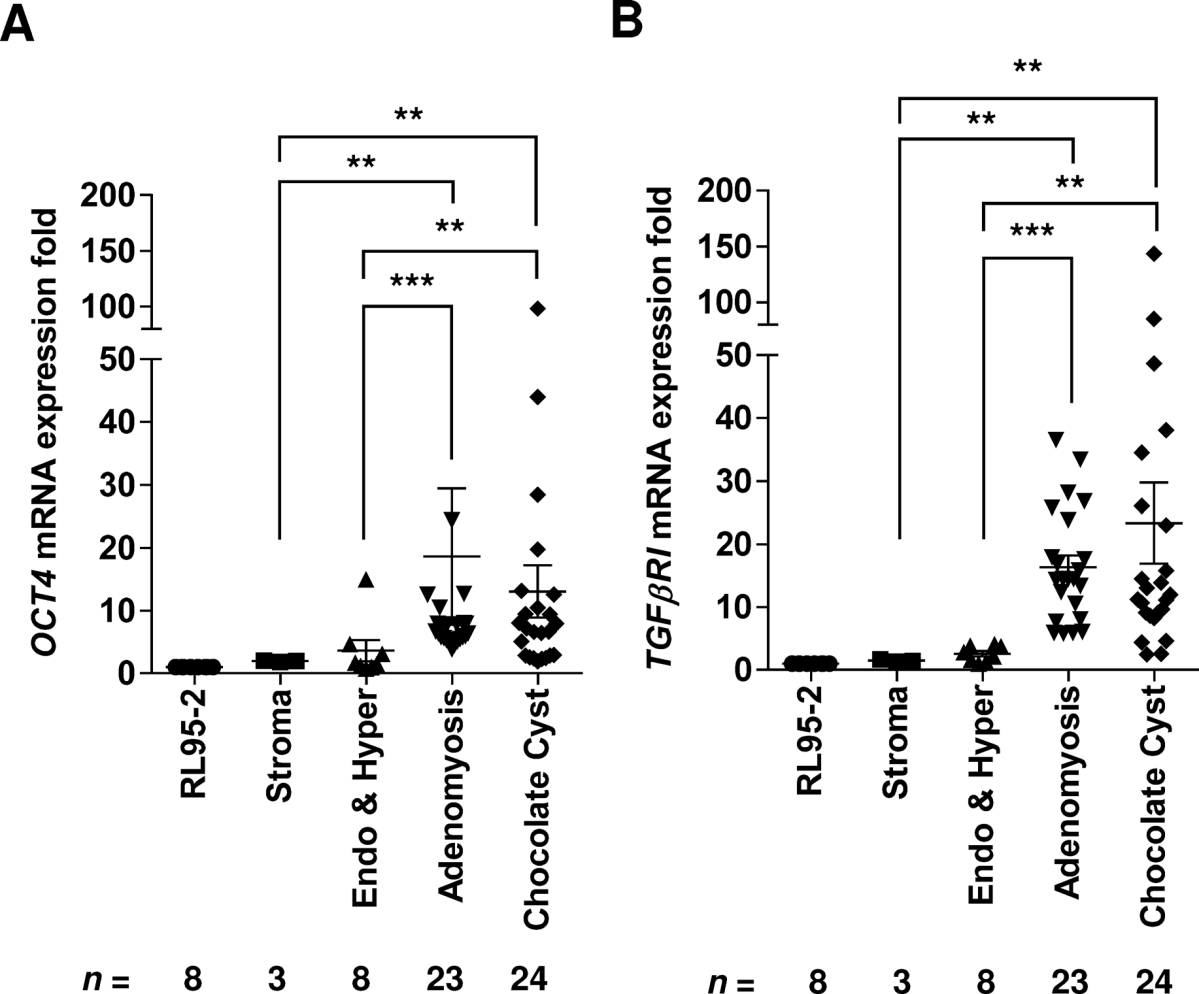
Quantification of mRNA levels of *OCT4* and TGF-β receptor I (*TGF-β RI*) in human endometrial tissues. Reverse transcription and quantitative real-time PCR were used to quantify the levels of **(A)** *OCT4* and **(B)** *TGF-β RI* mRNAs in RL95-2 human endometrial cell lines, primary endometriotic stromal cells, normal endometrium and hyperplastic endometrium (*n* = 8), adenomyosis (*n* = 23), and chocolate cysts (*n* = 24). The RL95-2 cells were used as internal controls for normalization. Results are expressed as mean ± SD. **P < .01, ***P < .001 in comparison with the control group of normal and hyperplastic endometrium tissues. By Mann-Whitney-U test.

The gene expression levels of *OCT4* and *TGF-β RI* were analyzed by qRT-PCR and were compared between the tissue samples from low-migratory normal and hyperplastic endometrium and the tissue samples from high-migratory adenomyosis and chocolate cyst tissues. As shown in Fig 1A, the *OCT4* mRNA expression levels in the adenomyosis and chocolate cyst samples (ectopic endometria) were higher than those in the normal endometrium and eutopic hyperplastic endometrial samples (*P* < .001 for adenomyosis and *P* < .01 for chocolate cyst). In addition, the *TGF-β RI* expression levels were higher in the ectopic tissues than those in the control tissues (*P* < .001 for adenomyosis and *P* < .01 for chocolate cyst, Fig 1B). These results demonstrated the significant increase of mRNA levels of *OCT4* and *TGF-β RI* in ectopic endometriotic tissues.

### Positive correlations of mRNA levels among *OCT4* and *TGF-β RI* and migration-related genes in human endometriotic tissues

**Fig 2:**
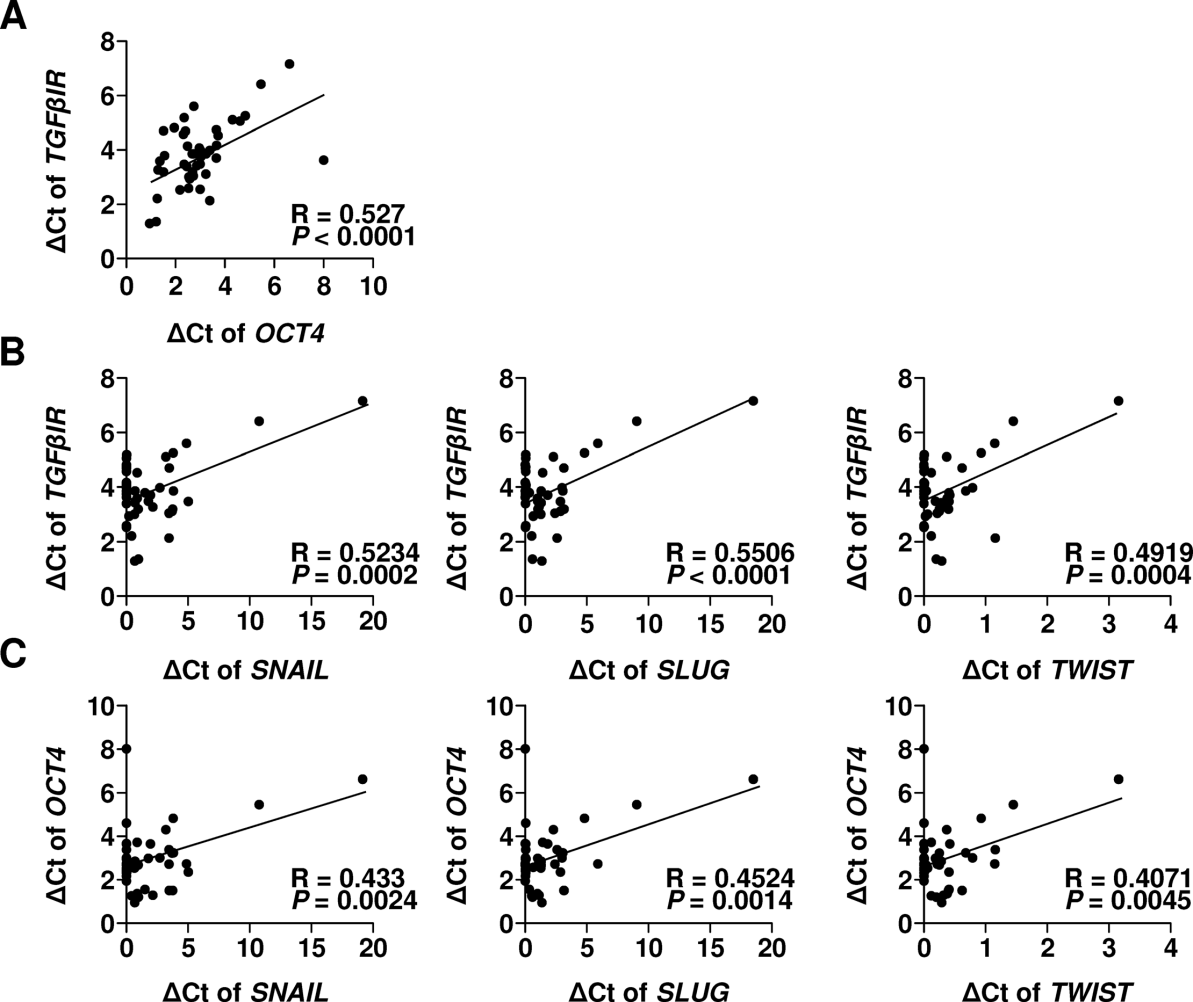
Positive correlations between the expression of *TGF-β RI* and *OCT4* and that of migration-related genes in human endometriotic tissues. The mRNA levels in endometriotic tissue samples obtained from patients with adenomyosis (*n* = 23) and chocolate cysts (*n* = 24) were quantified using reverse transcription and quantitative real-time PCR. The transcriptional levels (ΔCt of gene levels/β2-microglobulin) of **(A)** *OCT4*, or **(B)** migration-related genes of *SNAIL*, *SLUG*, or *TWIST*, were compared with those of *TGF-β RI*, and **(C)** The transcriptional levels (ΔCt of gene levels/β2-microglobulin) of *SNAIL*, *SLUG*, or *TWIST* were compared with those of *OCT4*, using the Pearson correlation analysis to determine the statistical correlations.

To examine the correlations among the mRNA levels of *OCT4*, *TGF-β RI*, and migration-related genes, ectopic chocolate cyst and adenomyosis samples were collected and analyzed for mRNA expression levels of *OCT4*, *TGF-β RI*, *SNAIL*, *SLUG*, and *TWIST*, using the qRT-PCR method. As shown in Fig 2, there was a positive correlation between *TGF-β RI* and *OCT4* (Fig 2A, R = 0.527, *P* < .0001), *TGF-β RI* and the migration-related genes (Fig 2B, R = 0.5234, *P* = 0.002 for *SNAIL*, R = 0.5506, *P* < .0001 for *SLUG*, and R = 0.4919, *P* = 0.0004 for *TWIST*), and *OCT4* and the migration-related genes (Fig 2C, R = 0.433, *P* = 0.0024 for *SNAIL*, R = 0.4524, *P* < .0014 for *SLUG*, and R = 0.4071, *P* = 0.0045 for *TWIST*) regarding the mRNA levels. Given the fact that serum TGF-β levels are up-regulated in patients with endometriosis [19, 21, 22], the positive correlations among *OCT4*, *TGF-β RI*, and migration-related genes suggested that TGF-β/TGF-β RI may play a crucial role in the transcriptional regulation of *OCT4* and migration-related genes in human endometriotic tissues.

### TGF-βI increases the expressions of OCT4 and SNAIL and N-CADHERIN (N-CAD) in human endometriotic cells

**Fig 3:**
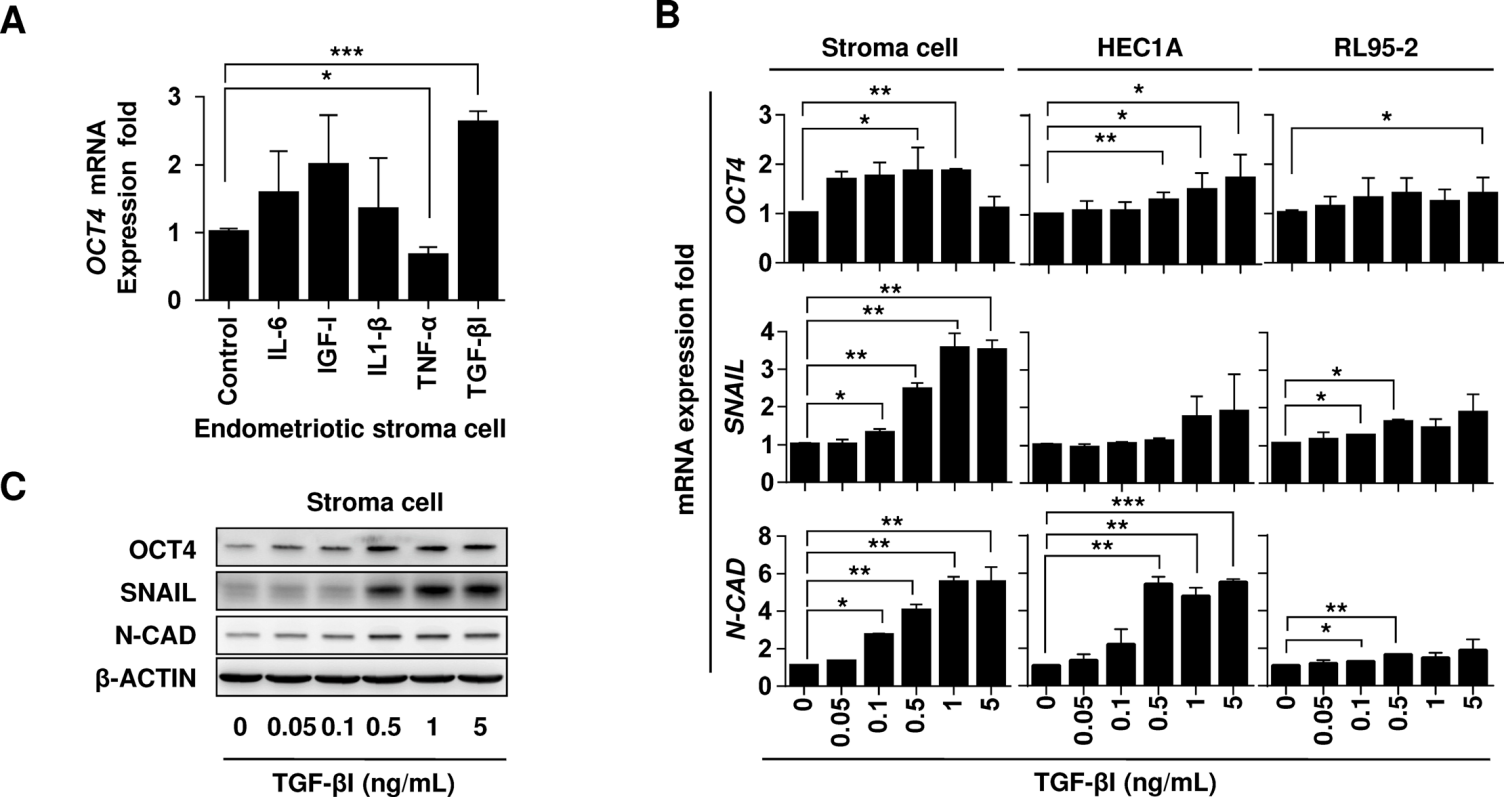
TGF-βI increases the expression levels of OCT4 and migration-related genes in human endometrial cells. **(A)** The relative expression folds of OCT4 mRNA in human endometriotic stromal cells under different cytokine or growth factor treatments were normalized to the untreated control group. The primary human endometriotic stromal cells were treated with IL-6 (50 ng/mL), IGF-I (50 ng/mL), IL-1β (50 ng/mL), TNF-α (50 ng/mL), and TGF-βI (1 ng/mL) for 24 hours. **(B)** The relative transcriptional levels of *OCT4*, *SNAIL*, and *N-CADHERIN (N-CAD)* in the RL95-2, HEC1A, and primary endometriotic stroma cells under TGF-β treatment (0, 0, 05, 0.1, 0.5, 1, and 5 ng/mL) were determined using quantitative real-time PCR. Data are representative of at least 3 independent experiments. **(C)** The relative protein levels of primary human endometriotic stromal cells in (B) were analyzed using Western blot analysis. *P < .05, **P < .01, and ***P < .001 by *t* test.

Inflammatory cytokines and growth factors have been associated with human endometriosis [18–20]. To determine the potential growth factor/cytokine that regulates OCT4 expression, the primary human endometriotic stromal cells were used as an *in vitro* model for treatment with IL-6, IGF-I, IL-1β, TNF-α, and TGF-βI, and the *OCT4* mRNA levels were examined using qRT-PCR. As shown in Fig 3A, when compared with other growth factors or cytokines, TGF-βI most significantly increased the *OCT4* mRNA levels in the endometriotic stromal cells (*P* < .001). To further examine the role of TGF-βI in the expressions of OCT4 and migration-related genes, as currently there is no appropriate endometrium cell line being available, three cell lines of RL-95-2 (epithelial carcinoma), HEC1A (epithelial adenocarcinoma), and primary stromal cells (mesenchymal chocolate cyst) were used to treat with increasing dose of TGF-βI (0, 0.05, 0.1, 0.5, 1, and 5 ng/mL). The gene and protein expression levels of OCT4 and migration-related genes were detected using qRT-PCR and western blotting, respectively. Fig 3B showed that the TGF-βI treatment dose-dependently increased the mRNA levels of *OCT4* and the migration-related genes *SNAIL* and *N-CAD* in human endometriotic stromal cells. The HEC1A and RL95-2 cell lines, with an epithelial cell origin, also exhibited trends of increasing gene expressions of *OCT4*, *SNAIL*, and *N-CAD* with increasing TGF-βI treatment dosages (Fig 3B). The effect of TGF-β on the protein expressions in human endometriotic stromal cells were further supported by western blotting analysis. As shown in Fig 3C, The TGF-βI dose-dependently increased the protein levels of OCT4 and the migration-related genes of SNAIL and N-CAD in human primary endometriotic stromal cells.

### Pluripotent transcription factor OCT4 mediates TGF-βI-induced SNAIL/N-CAD expression and cell migration in human endometriotic cells

**Fig 4:**
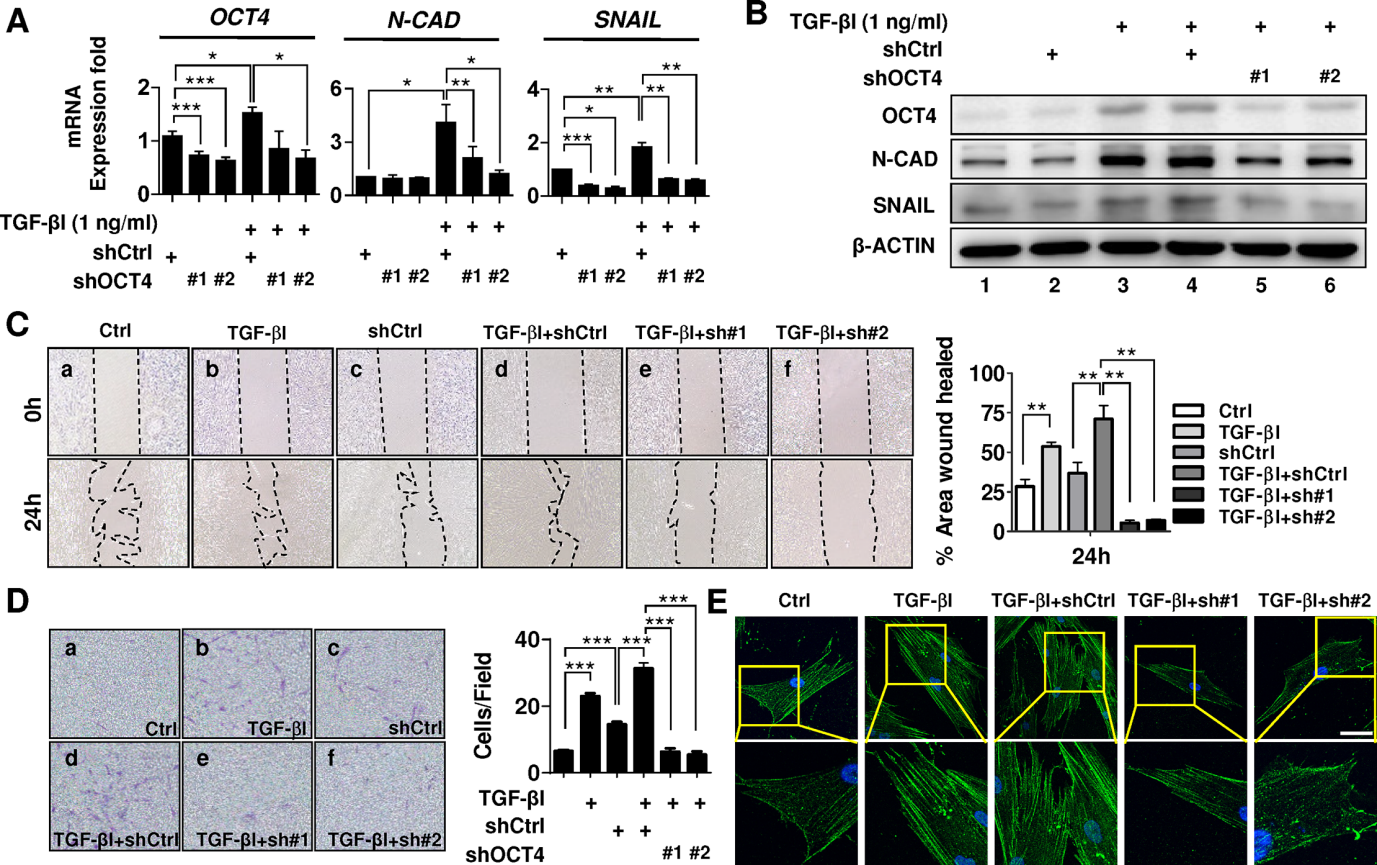
Pluripotent transcription factor OCT4 mediates TGF-βI-induced N-CAD/SNAIL expressions and cell migration in human endometriotic cells. **(A)** The effects of OCT4 knockdown on the TGF-βI-induced gene expression levels of *OCT4*, *N-CAD*, and *SNAIL*. **(B)** The effects of OCT4 knockdown on the TGF-βI-induced protein expression levels of OCT4, N-CAD, and SNAIL. shCtrl, control shRNA; sh#1 and sh#2, shOCT4. **(C)** The effects of OCT4 knockdown on the TGF-βI-induced cell migration of human endometrial cells at 0 and 24 hours was evaluated using a wound-closure assay. The figure presents the proportion of the areas in the wound-closure assay under different experimental conditions. **(D)** The effects of OCT4 knockdown on the TGF-βI-induced cell motility of human endometrium cells at 24 hours was quantified using a transwell assay. **(E)** The cellular localization of actin in the human primary endometriotic stromal cells with or without OCT4 silencing was evaluated using immunocytochemical staining. The nuclei were stained with DAPI (blue), and actin was detected with FITC-conjugated secondary Ab (green). High-magnification images are presented. shCtrl, control shRNA; #1 and #2, shOCT4. Bar = 25 μm. *P < .05, **P < .01, and ***P < .001 by *t* test.

To examine the role of the pluripotent transcription factor OCT4 in TGF-βI-induced expression of migration-related genes and endometrial cell migration, the primary human endometriotic stromal cells were transfected with either shOCT4 to generate OCT4-knockdown cells or control plasmids to produce control cells. The mRNA levels of *OCT4* and the migration-related genes *N-CAD*/*SNAIL* evaluated using qRT-PCR were compared between cells with or without TGF-βI treatment. The TGF-βI-treated primary endometriotic stromal cells exhibited significantly increased mRNA levels for *OCT4* and the migration-related genes *N-CAD* and *SNAIL* (Fig 4A). Knockdown of OCT4 significantly decreased the *N-CAD* and *SNAIL* mRNA levels. These results were further supported by western blot analysis (Fig 4B). Experiments performed on epithelial endometrium cell lines, RL95-2 and HEC1A provided additional support for the role of OCT4 in TGF-βI-induced expressions of migration-related genes (S1 Fig). These results suggested that TGF-βI may regulate the N-CAD and SNAIL protein expression through OCT4 in human endometriotic stromal cells.

To determine whether OCT4 mediates TGF-βI stimulated cell migration, the primary endometriotic stromal cells transfected with either shCtrl or shOCT4 was treated with or without TGF-βI. Results from the wound-closure assay (Fig 4C) and transwell assay (Fig 4D) revealed that TGF-βI treatment significant increased cell migration ability when compared with the control group (*P* < .01 for wound-closure assay; and *P* < .001 for transwell assay). On the contrary, knockdown of OCT4 significantly decreased the TGF-βI-induced migratory activity (*P* < .01 for wound-closure assay and *P* < .001 for transwell assay) (Fig 4C and 4D). The cell proliferation under different experimental conditions in 24 h was shown in S2 Fig. Moreover, immunocytochemical analysis and confocal imaging revealed that knockdown of OCT markedly suppressed the TGF-βI-induced intracellular actin fiber extension and cell morphology change in the human endometriotic stromal cells when compared with the control group (Fig 4E). In summary, our results demonstrated a crucial role of the pluripotent transcriptional factor OCT4 in TGF-βI-stimulated cell migration in human endometrial cells.

## Discussion

Endometriosis is a nonmalignant disorder with the presence of ectopic endometrial tissues, such as adenomyosis and chocolate cyst [1]. It affects approximately 10% of women of reproductive age and is associated with chronic pelvic pain, dysmenorrhea, and infertility [36]. To date, studies on the endometriosis development have addressed the changes between the eutopic and ectopic endometrium tissues, but limited studies have evaluated the role of the growth factors in peritoneum fluids, such as TGF-β, in the development of endometriosis.

Several studies have hypothesized the role of TGF-β in the development of endometriotic lesions based on the presence of high levels of *TGFβI* mRNA and TGFβI protein in the endometriotic lesions, peritoneal fluid, and serum in patients with endometriosis [19, 21, 22]. In a previous study, TGF-β-null females developed fewer and smaller peritoneal endometriosis lesions than did wild-type counterparts in a mouse endometriosis model [35]. Although TGF-βI/TGF-β RI is hypothesized to induce the development of endometriosis by activating cell migration, the understanding of TGF-β/TGF-β RI and stemness-related gene OCT4 in endometriosis remains to be elucidated. TGF-β is upregulated in the peritoneal fluid of endometriosis patients [22], and our results revealed high TGFβ-RI expression levels in the endometriosis tissue. Because of the higher TGF-β concentration in endometriotic peritoneal fluid, we treated the human endometriotic cells with a higher dose of TGF-β to demonstrate the TGF-β effect on the increasing expression of pluripotent transcription factor OCT4, thereby resulting in stimulated cell migration in human endometriotic cells.

Recently, studies have reported the re-expression of the pluripotent transcription factor OCT4 in somatic tissues [37, 38], malignant tumors [39–41], and ectopic endometrial tissues [12, 15, 16]. The upregulation of *OCT4* transcription in somatic tissues has been associated with the initiation of cancer stemness and metastasis [42, 43]. Emerging evidence suggests that tumor metastasis is strongly influenced by EMT which is a hallmark sign of cancer stemness [44]. Studies have demonstrated that the transcription factors TWIST, SNAIL, and SLUG regulate EMT and increase cell motility [44–47]. In support of the stemness and EMT, our previous report demonstrated a positive correlation between the expression levels of the *OCT4* and EMT-related genes in ectopic endometriotic tissues, and the forced OCT4 expression promotes the cell migration in human endometriosis [15]. In advance of the previous findings, the present study results further demonstrated that there is a significant positive correlation of *OCT4* with *TGF-β RI* and *TGF-β RII* (Fig 1 and S3 Fig); and the aberrant upregulation of TGF-βI/TGF-β RI in human endometriotic cells increases cell migration through the induction of OCT4 expression (Figs 3 and 4). The OCT4 expression in ectopic endometrial cells, particularly in response to TGF-βI/TGF-β RI signaling, may facilitate self-renewal and increase cell migration, initiating the development of endometriosis. This hypothesis is strongly supported by the recent findings that the risk of ovarian cancer was associated with aberrant TGF-β signaling [48].

OCT4 expression is known to be regulated by HIF-2-alpha transcription factor [49], EpCAM transmembrane glycoprotein [50], growth factor of insulin-like growth factor I (IGF-I) [51], and steroid hormone estrogen [52]. In the present study, we further demonstrated that TGF-βI stimulates the OCT4 expression, and the OCT4 expressed in the endometriotic stromal cells, in turn, mediated the TGF-βI/TGF-β RI -induced cell migration. In addition to TGF-β, IGF-I is another serum marker for human endometriosis [53]. Reportedly, patients with late-stage endometriosis exhibited significantly higher IGF-I levels than did those with early-stage endometriosis and healthy control participants, which suggests that IGF-I is a crucial mediator of the development and maintenance of endometriosis or its progression to an advanced-stage disease [53]. In support, our results showed that IGF-I tends to increase the OCT4 mRNA levels in human endometriotic stromal cells (Fig 3A). In consistence with these results, our previous study conducted using germline stem cells, demonstrated that the OCT4 expression was regulated by an IGF-IR-HIF-2α signal loop in pluripotent mouse germline stem cells [54] and a IL-6-IGF-IR signal in HBV-related hepatocellular carcinoma [55]. These results strongly support a crucial role of OCT4 in the niche growth factor-mediated development of endometriotic lesions.

In conclusion, this study provides new evidence regarding the effects of the pluripotent transcription factor OCT4 in the TGF-βI/TGF-β RI -associated pathophysiology of ectopic endometrial cell migration. These findings may be useful for developing therapeutic strategies to prevent ectopic endometriosis by targeting TGF-β-OCT4 signaling in the future.

## Supporting Information

S1 Fig Effects of OCT4 on TGF-βI-induced mRNA expression levels of *OCT4*, *N-CAD*, and *SNAIL* in human endometrial RL95-2 and HEC1A cell lines. Quantitative real-time PCR analysis was independently repeated at least 3 times. The relative mRNA expression folds of *OCT4*, *N-CAD*, and *SNAIL* in TGF-βI-treated human endometrial RL95-2 and HEC1A cells with or without OCT4 silencing (shOCT4). shCtrl, control shRNA; #1 and #2, shOCT4. *P < .05, **P < .01, and ***P < .001 by *t* test.
(TIFF)

S2 Fig Effects of TGF-β and shRNA on cell-proliferation in primary endometriotic stromal cells. The viability of human endometriotic stromal cells with or without shRNA and/or TGF-β (1 ng/ml) treatment for 24h was evaluated using a WST-1 assay. The cell viability at 24h is represented with folds when compare to that at 0 h incubation time. Three independent experiments were performed for each experimental condition.
(TIFF)

S3 Fig Positive correlations between the expression of *TGF-β RII* and *OCT4* in human endometriotic tissues. The transcriptional levels (ΔCt of gene levels/β2-microglobulin) of *OCT4* were compared with those of the *TGF-β RII* using the Pearson correlation analysis to determine the statistical correlations.
(TIFF)

S1 Table Description of the study population. (PDF)

S2 Table Real-time quantitative PCR primer and product size. (PDF)

S3 Table Antibodies list. (PDF)
