# Supplementary figures and images for: TGF-βI Regulates Cell Migration through Pluripotent Transcription Factor OCT4 in Endometriosis
Source: PLoS One. 2015 Dec 16;10(12):e0145256. doi: 10.1371/journal.pone.0145256 (PMC4682958; doi:10.1371/journal.pone.0145256)

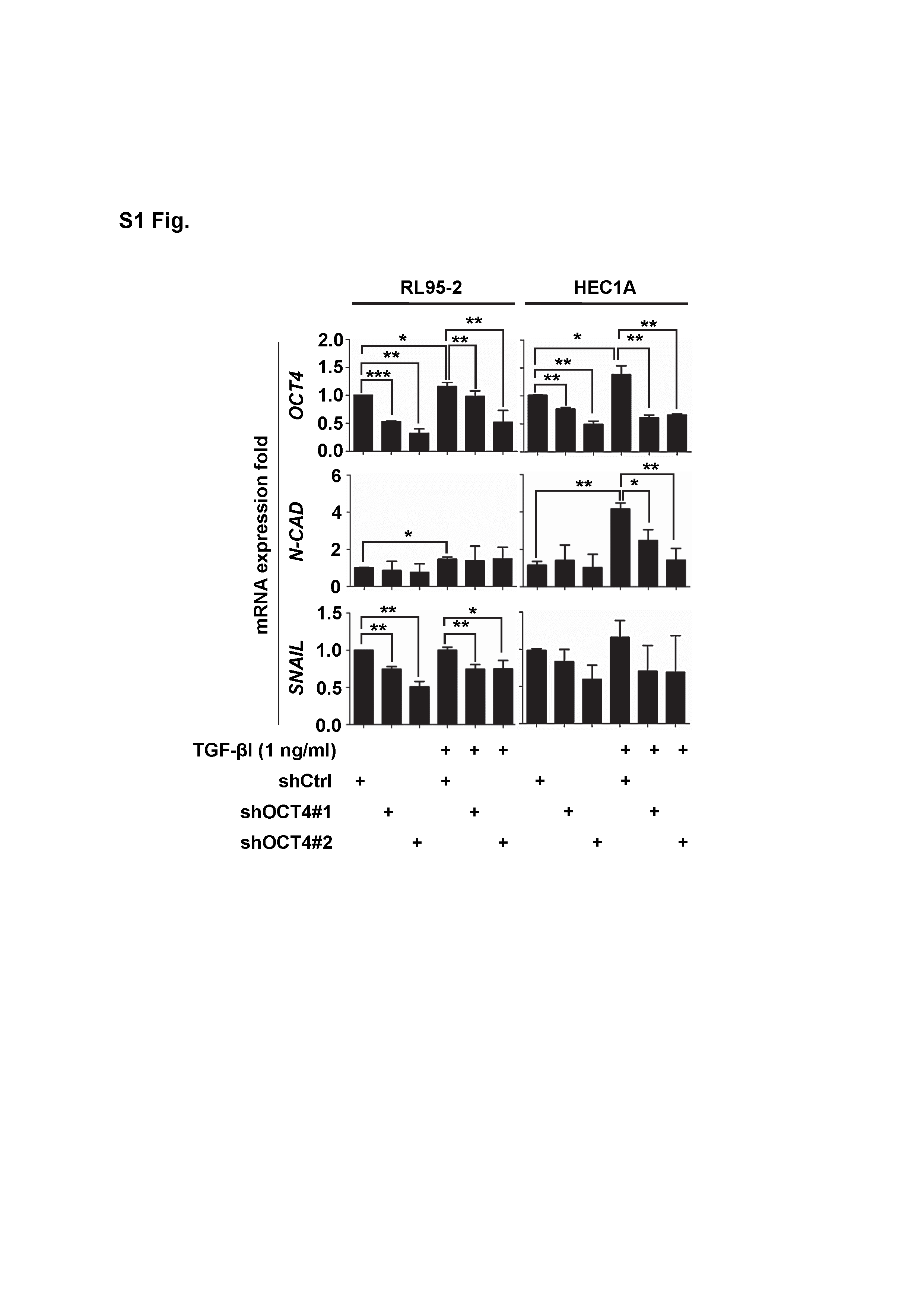

Supplement: S1 Fig — Quantitative real-time PCR analysis was independently repeated at least 3 times. The relative mRNA expression folds of OCT4, N-CAD, and SNAIL in TGF-βI-treated human endometrial RL95-2 and HEC1A cells with or without OCT4 silencing (shOCT4). shCtrl, control shRNA; #1 and #2, shOCT4. *P < .05, **P < .01, and ***P < .001 by t test. (TIFF) [file pone.0145256.s001.tiff]

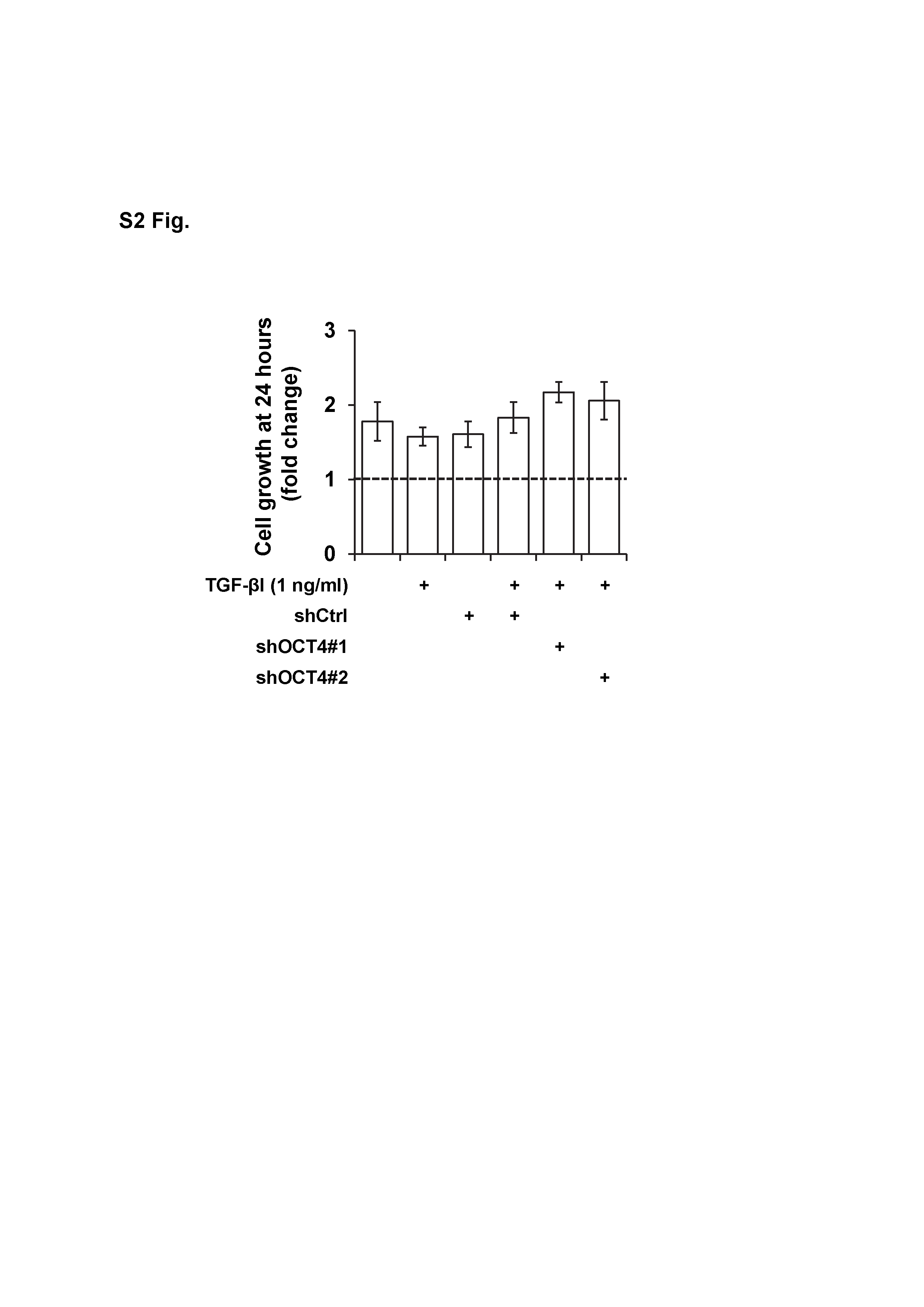

Supplement: S2 Fig — The viability of human endometriotic stromal cells with or without shRNA and/or TGF-β (1 ng/ml) treatment for 24h was evaluated using a WST-1 assay. The cell viability at 24h is represented with folds when compare to that at 0 h incubation time. Three independent experiments were performed for each experimental condition. (TIFF) [file pone.0145256.s002.tiff]

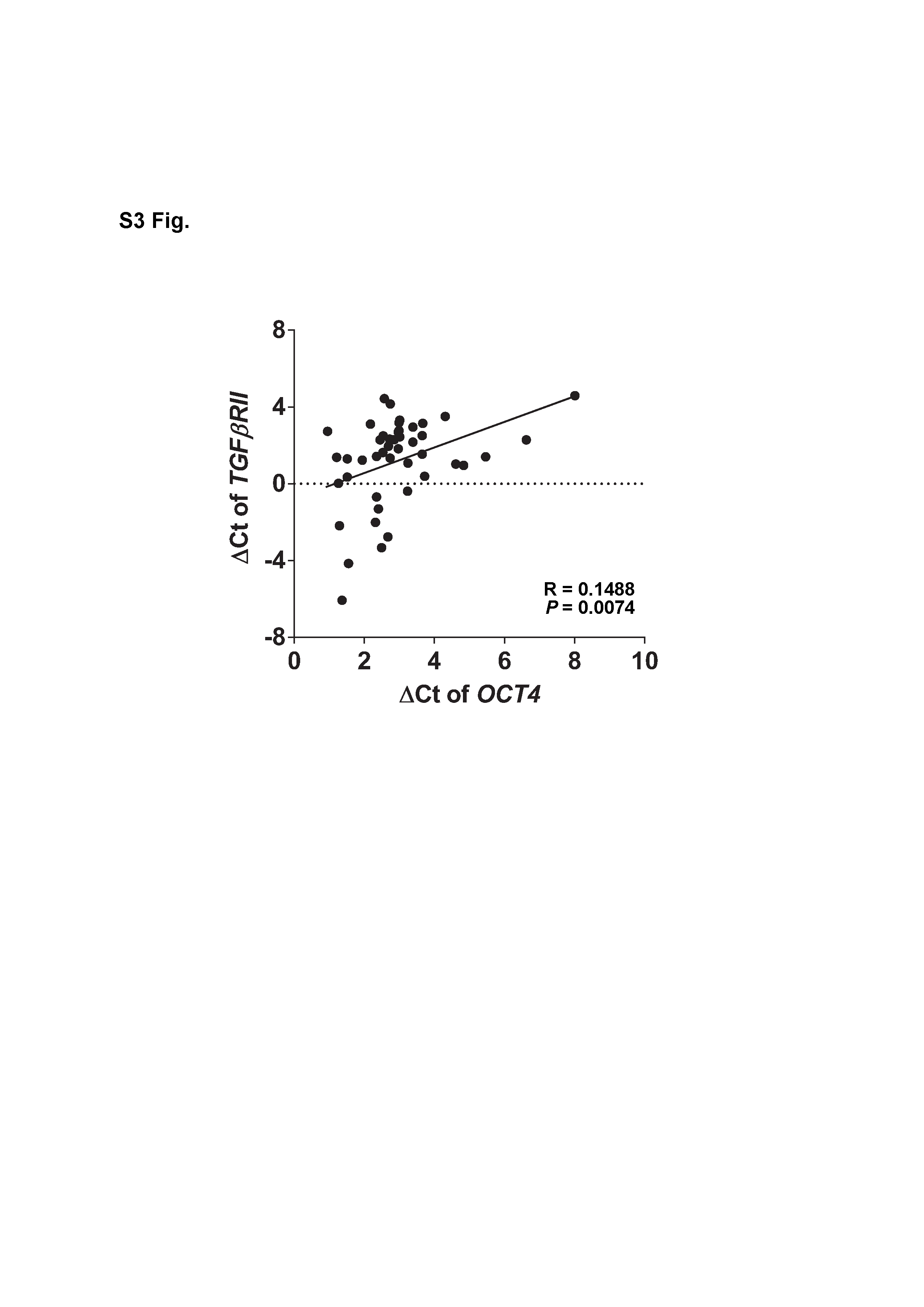

Supplement: S3 Fig — The transcriptional levels (ΔCt of gene levels/β2-microglobulin) of OCT4 were compared with those of the TGF-β RII using the Pearson correlation analysis to determine the statistical correlations. (TIFF) [file pone.0145256.s003.tiff]
