# Supplementary material for: TGF-βI Regulates Cell Migration through Pluripotent Transcription Factor OCT4 in Endometriosis
Source: PLoS One. 2015 Dec 16;10(12):e0145256. doi: 10.1371/journal.pone.0145256 (PMC4682958; doi:10.1371/journal.pone.0145256)
Supplement: S1 Table — (PDF) [file pone.0145256.s004.pdf]

S1 Table Description of the study population

	Endometrium (Normal and Hyperplasia) (*n* = 8)	Endometriosis (Adenomyosis and Chocolate cyst) (*n* = 47)	*P*
**Age (y)**	46.4 ± 5.8 (37-54)	38.7 ± 6.9 (26-58)	**0.005**[*a]
**BMI (kg/m^2)**	24.6 ± 4.5 (20-31.3)	22.7 ± 3.3 (18.1-31.6)	0.175[a]
**Age at menarche (y)**	12.6 ± 0.7 (12-14)	13.0 ± 1.6 (11-17)	0.532[a]
**Cycle length (d)**	29.0 ± 1.1 (28-30)	28.0 ± 2.6 (20-33.5)	0.292[a]
**CA125 (U/mL)**	19.7 ± 6.9 (15.6-32.2)	65.5 ± 61.8 (5.2-284.7)	**0.040**[*a]
**Regular cycles (%)**	8 (100%)	45 (95.7%)	0.552[b]
**Smoker (%)**	1 (12.5%)	3 (6.4%)	0.538[b]
**Dysmenorrhea (%)**	5 (62.5%)	33 (70.2%)	0.663[b]
**Married or living with partner (%)**	7 (87.5%)	41 (87.2%)	0.983[b]

Note: Continuous variables are presented as mean ± SD (range), while categorical variables are presented as n (%).

[a]Independent t-test

bChi-squared test
