# Supplementary material for: TGF-βI Regulates Cell Migration through Pluripotent Transcription Factor OCT4 in Endometriosis
Source: PLoS One. 2015 Dec 16;10(12):e0145256. doi: 10.1371/journal.pone.0145256 (PMC4682958; doi:10.1371/journal.pone.0145256)
Supplement: S2 Table — (PDF) [file pone.0145256.s005.pdf]

**S2 Table Real-time quantitative PCR primer and product size**

| Gene | Accession | Forward primers | Reverse primers | Product size (bp) |
|---|---|---|---|---|
| *TGF-βRI* | NM_004612 | 5'-GGCTTTTCTCCACATGCTTAGG-3' | 5'-GGCAACAGAGATCACCTGTAGACA-3 | 78 |
| *OCT4* | NM_002701 | 5'-CAACTCCGATGGGGCCT-3' | 5'-CTTCAGGAGCTTGGCAAATTG-3' | 148 |
| *SNAIL* | NM_005985 | 5'-CTTCCAGCAGCCCTACGAC-3' | 5'-CGGTGGGGTTGAGGATCT-3' | 70 |
| *N-CADHERIN* | NM_001792 | 5'-GGTGGAGGAGAAGAAGACCAG-3' | 5'-GGCATCAGGCTCCACAGT-3' | 72 |
| *SLUG* | NM_003068 | 5′-GAGCATTTGCAGACAGGTCA-3′ | 5′-CCTCATGTTTGTGCAGGAGA-3′ | 123 |
| *TWIST* | NM_000474 | 5′-TCTCGGTCTGGAGGATGGAG-3′ | 5′-GTTATCCAGCTCCAGAGTCT-3′ | 152 |
| *B2M* | NM_004048 | 5'-GATGAGTATGCCTGCCGTGTG-3' | 5'-CAATCCAAATGCGGCATCT-3' | 114 |
| **Gene** | **Accession** | **Clone ID** | **Target sequence** | |
| shCtrl | | TRCN0000072226 | 5'-CGATCGTAATCACCCGAGTGT-3' | |
| shOCT4＃1 | NM_002701 | TRCN0000004879 | 5'-TCATTCACTAAGGAAGGAATT-3' | |
| shOCT4＃2 | NM_002701 | TRCN0000004881 | 5'-CCCTCACTTCACTGCACTGTA-3' | |
