# Supplementary material for: TGF-βI Regulates Cell Migration through Pluripotent Transcription Factor OCT4 in Endometriosis
Source: PLoS One. 2015 Dec 16;10(12):e0145256. doi: 10.1371/journal.pone.0145256 (PMC4682958; doi:10.1371/journal.pone.0145256)
Supplement: S3 Table — (PDF) [file pone.0145256.s006.pdf]

S3 Table Antibodies list

Protein	Assay	Ab Cat. No.	Company	Origin	Dilution	Incubation period
OCT4A	WB[1]	#2907	Epitomics	Rabbit	1:1000	overnight, 4°C
Snail	WB	#3895	Cell Signaling	Mouse	1:1000	overnight, 4°C
N-cadherin	WB	#2019	Epitomics	Rabbit	1:3000	overnight, 4°C
β-Actin	WB	A5441	Sigma-Aldrich	Mouse	1:10000	overnight, 4°C
β-Actin	ICC[2]	A5441	Sigma-Aldrich	Mouse	1:200	2 h, RT[3]

[1]WB, western blot. [2]ICC, immunocytochemistry. [3]RT, room temperature.
